# Views of teenagers on termination of pregnancy at Muyexe high school in Mopani District, Limpopo Province, South Africa

**DOI:** 10.4102/phcfm.v8i2.945

**Published:** 2016-05-31

**Authors:** Nditsheni J. Ramakuela, Tsakani R. Lebese, Sonto M. Maputle, Lindiwe Mulaudzi

**Affiliations:** 1 Department of Advanced Nursing Science, School of Health Sciences, University of Venda, South Africa; 2 Department of English, School of Human and Social Sciences, University of Venda, South Africa

## Abstract

**Background:**

Teenage pregnancy is a global social health concern especially because of the HIV and AIDS pandemic, sexually transmitted infections, high rate of termination of pregnancy (TOP), adolescents’ parenthood and decreased level of contraceptives.

**Aim:**

To explore the views of teenagers on the TOP at Muyexe high school in a rural village of Mopani District, Limpopo Province.

**Setting:**

Muyexe high school in a rural village of Mopani District, Limpopo Province, in South Africa.

**Methodology:**

A qualitative method using explorative and descriptive designs was used to find in-depth description and understanding of teenagers’ views on TOP. The target population was girls aged 15–19 years at Muyexe high school in Mopani District. Non-probability, convenient sampling was used to select high school teenage girls who had undergone TOP for the study. Data were collected using individual self-report technique (interview). Tesch’s eight steps of qualitative data analysis were used. Measures to ensure trustworthiness and ethical considerations were observed.

**Results:**

Two major themes were revealed: (1) Views of teenagers regarding TOP (poverty, relationship problems and single parenthood, negative impact on the teen’s life while attending school) and (2) teenager’s fears regarding pregnancy (stigma, fear of parents and friends, rape and incest and fear of giving birth).

**Conclusion:**

Majority of participants had knowledge about TOP; some had experiences about TOP while others held inadequate knowledge. Recommendations were based on the findings by teaching dangers of TOP and various contraceptive methods to prevent unwanted pregnancies and TOP.

## Introduction

South Africa, like many other countries, is experiencing a high rate of youth pregnancy and its associated social ills, regardless of free contraceptive services. In rural areas, the rate is significantly higher as evidenced by the number of youth who fall pregnant at any given point; hence, there is poor utilisation of contraceptive services in rural communities. The World Health Organisation^1^ estimated that close to 17 million girls under the age of 20 years give birth annually and a further 4.4 million abortions were sought by adolescents each year worldwide as a result of inadequate or poor utilisation of contraceptives.

The Abortion Reform Action Group created momentum for abortion reform in South Africa in 1975.^2^ The *Abortion Act* later became the *Abortion and Sterilization Act* that granted greater freedom to women seeking abortions. Under this Act, abortions could be performed legally only when a pregnancy could seriously threaten a woman’s life or her physical or mental health, could cause severe handicap to the child or was the result of rape (which had to be proved), incest or other unlawful intercourse, such as with a women with a permanent mental handicap. On December 11, 1996, the South African government enacted the choice on *Termination of Pregnancy Act* No. 2 of 1975.^3^ This gives women of any age or marital status access to abortion service. One of the features of the Act is the respect for women’s right to choose, irrespective of the age of a pregnant woman.^2^

Schuster, Bell, Berry and Kanouse^4^ indicated that teenagers still lack knowledge about the advantages and disadvantages of termination of pregnancy (TOP) and the use of contraceptives to prevent unwanted pregnancies that lead to TOP and the consequences thereof. Many teenagers view TOP as a way of preventing rejection from parents, stigma from other peers and because they want to further their education. Some do it because they are rejected by their partners while others feel they are not ready to become mothers. Teenagers think they cannot afford to raise a child and are scared of the impact of motherhood in their lives because they are still teenagers. Other teenagers perform TOP as a method of birth control. According to Guttmacher,^2^ approximately 68 000 teenagers die annually as a result of complications of unsafe TOP. However, between 2 and 7 million teenagers each year survive unsafe TOP but sustain long-term damage or disease (such as incomplete TOP, infection because of sepsis, haemorrhage and injury to the internal organs such as puncturing or tearing of the uterus). They also concluded that TOP is safer in countries where it is legal, but dangerous in countries where it is outlawed and performed clandestinely.

In Nigeria, teenagers view TOP as an immediate solution to unplanned pregnancy. Teenagers opt to TOP because they do not want interference with schooling, not being old enough to get married, fear of family members knowing, not planning to marry the partner, being jilted by a fiancé, following rape or incest and not knowing the actual father.^5^

Researchers found that there is a high rate of TOP among teenagers as teenage pregnancy in South Africa rose from a low of 2.0% among 15-year olds and peaked at 30.2% among 19-year olds in 2002.^6^ Data from the 2003 RHRU survey show that in South Africa, teenagers aged 17–19 account for 93.0% of teenage pregnancy and majority opted for TOP.^7^ The high South African TOP rate between 1997 and 2012 was 2.7% – 7.7%, while in Limpopo Province, it rose from 0.6% in 1997 to 6.6% in 2012.^8^ Five teenagers visit a TOP clinic daily requesting abortion while some of them return several times (e.g. twice in 6 months) for it. Legalisation of TOP as a free service for women in South Africa has led many teenagers to terminate pregnancy irrespective of the conditions, for example, rape, congenital defects of the baby and the consequences. There are many facilities that are regulating TOP for free or for a fee with implications, such as inability to conceive or death of the teenager.

## Methodology

### Design

A qualitative research approach was chosen for this study to gain insight through discovering meaning and attempting to discover the depth and complexity of a phenomenon regarding teenagers’ views on the TOP.^9^

### Population and sampling

The population were 25 girls aged 15–19 years from Muyexe High School in Mopani District. The target population consisted of teenagers aged 15–19 years from Muyexe high school in Mopani District, while the accessible population was that portion or part of the target population to which the researcher had reasonable access and who came to the arranged venues to form part of the interviews.^9,10,11^ A non-probability, convenient sampling technique was used to select participants for the study. The first 25 teenagers to enter the classroom were conveniently selected, but 19 participants were interviewed as saturation was reached.^12^ Criteria for inclusion in the study were teenage girls aged 15–19 years and who had had an abortion.

### Data collection methods

Data were collected by the researcher using self-report technique by interviewing teenagers individually for 45 min to an hour using semi-structured interviews to find what they believe, think or know regarding TOP. Interview dates were arranged with the contact person, and data were collected in a classroom of the school in Xitsonga. Data were collected during the day from 14:00 after school for about 2 h a day for 7 days. Tape recorder and field notes were used during data collection. Data collection continued until saturation was reached (19th participant). Data collected were then transcribed verbatim from Xitsonga to English by a language expert.^9^

### Data analysis

Tesch’s open method was used to analyse data. The researcher listened to audio tapes and read and re-read the verbatim transcripts to get a global understanding of the interviews and familiarise her and/or himself with the data. Thereafter, the researcher randomly picked each verbatim transcript and started analysing them one by one, until all the transcripts were analysed and similar ideas or topics were coded from each category. A number of themes emerged.^13^ Data were analysed by transcribing the data that were gathered during interview. Coding was used to organise data collected in interviews and other types of documents by combining points which had similar meaning or related ideas and gave them sub-themes.

### Trustworthiness

The following measures were used to ensure trustworthiness, namely, credibility, dependability, transferability and confirmability. Credibility was ensured by staying in the field until data saturation was reached. That built trust and rapport between the researcher and the participants, which was needed in the gathering of data. It was achieved by interviewing teenagers who had already gone through TOP, and from this information researchers corrected obvious errors and provided additional information. Transferability was ensured by writing down all data collected in the participant’s language and transcribed in English. Dependability was ensured by pursuing interpretation in various ways and the researchers rephrased the question several times to check if the same information was obtained. Confirmability was ensured by the information provided by participants and not by researcher’s imagination, and the researchers used audio tape and field notes to record information.

### Ethical consideration

Permission to collect data was granted by the University of Venda and School higher degree committees, respectively. The Department of Education and the management of Muyexe high school also granted approval to continue with research. The chief was approached through written letters for permission to conduct the research in his village. An informed consent was obtained from each participant. This means that the aim, objectives, data collection method, duration and participation needed from the participant was explained and understood by each participant and made sure that each consent form was signed.^9^ Anonymity was ensured by not asking participants’ name or identity. Confidentiality was maintained by making sure that participants information was kept in a safe place and not exposed to other people. Privacy was also one of the most important priorities and was maintained by ensuring that the participant was alone, not watched by other people and in a safe environment during the interview. Researchers ensured voluntary participation to avoid harm.

## Results and discussion

Several views emerged from the study findings regarding TOP. The researcher used self-report technique interviewing teenagers individually for 45 min to an hour using semi-structured interviews. Recorded data were transcribed verbatim and all interviews generated the following themes: poverty and relationship problems and single parenthood, negative impact on the teens life, inadequate knowledge, fear of parents and friends, rape and incest, fear of giving birth and advantages and disadvantages of TOP.

The following themes and sub-themes as indicated in Table 1 emerged from the study.

**TABLE 1:** Themes and sub-themes.

Major themes	Sub-themes
Views of teenagers regarding termination of pregnancy	Poverty Relationship problems and single parenthood Negative impact on the teen’s life while attending school Lack of knowledge
Teenager’s fears regarding pregnancy	Stigma, fear of parents and friends Rape and incest Fear of giving birth

*Source*: Authors’ own work

### Theme 1: Views of teenagers regarding termination of pregnancy

From this theme, three sub-themes emerged, namely, poverty, relationship problems and single parenthood, and negative impact on the teen’s life while attending school.

#### Sub-theme 1.1: Poverty

TOP was introduced as a means of safely getting rid of unwanted pregnancy. However, teenagers had their own views regarding TOP as exemplified in the following excerpts:

‘I see poverty as a cause of terminating pregnancy due to poor family background wherein you will find that they depend on social grant of the grandparents’ she continued to say ‘you see if there is poverty in the family like financial problems and you are pregnant and still bring another burden, you tend to increase poverty and as a teen you opt for TOP.’ (Participant 1)

Poverty was a major factor in this study which led teenagers to opt for TOP with the reason that they were unable to take care of themselves and that to have a child was a burden upon them and their families. Teenagers felt bringing another burden to the family would worsen financial problems in their families as most of them depended on social grants of grandparents. This implied educating high school teens regarding teenage pregnancy.

Alcorn^14^ found that financial problems were also a challenge, causing poverty because of a lack of resources to cover the staggeringly high cost associated with pregnancy, birth and child rearing, especially if they do not have health insurance. Saving for a baby is one thing, but an unplanned pregnancy places an enormous financial burden on a teen who cannot afford to care for an infant. Teenagers in Europe view TOP as a way of preventing poverty because they are economically disadvantaged. Lack of adequate medical care during pregnancy places a newborn at a high risk for complications during birth and in early infancy.^15^

#### Sub-theme 1.2: Relationship problems and single parenthood

This was shared by another participant who said:

‘Denial and attitude by a partner can lead to TOP because they were not in a relationship they just engaged in sexual intercourse once and the boy feels that the baby is not his. This can really be frustrating on the part of a teen who is now standing alone to face the whole problem ahead of school interruption and being a single parent. Most teens do not have stable relationships as you are still young and not sure who is your Mr. right.’ (Participant 2)

The majority of female teenagers with unplanned pregnancies do not live with their partners or have committed relationships. These teenagers realised that in all likelihood they will be raising their child as single parent due to the denial by the partner and in some instance by their parents. Many are unwilling to take this big step because of interruptions of education or career, insufficient financial resources or inability to care for an infant because of care-giving needs of other children or family members.

Literature reveals that teens do not prefer to bring up children on their own because of their partners’ attitudes and would not be able to meet the needs of their children and their quality of life.^15^ Even in situations involving teens cohabitating with their partners, the outlook for unmarried teens as single mothers is discouraging; for teens in their 20s living with their partners at the time of birth, one-third ended their relationships within 2 years.^16^

#### Sub-theme 1.3: Negative impact on the teen’s life while attending school

This participant had this to say:

‘Teenagers choose to terminate pregnancy because they want to further their education, for example in other schools they don’t allow pregnant learners to attend classes whereas in other schools pregnant learners should come with their relatives to attend classes so that they can look after them in case of emergency.’ (Participant 6)

Pregnant learners were found to opt for TOP as pregnancy at their age interferes with learning and education. It implies that learners realise the lifelong impact of pregnancy when they have actually fallen pregnant themselves. Some schools were also found not to allow pregnant learners to attend school or required that they at least bring a parent to assist in cases of emergency.

Pregnancy that occurs in the wrong place at the wrong time can have a lifelong impact on a teenager’s ability to raise a child and earn a living. Less than half of teens who become mothers before the age of 18 graduate from high school, and college students who become pregnant are much less likely to complete their education than their peers.^17,18^ In Nigeria, teenagers view an immediate solution to an unplanned pregnancy by opting for TOP because they do not want interference with schooling.^5,19^

#### Sub-theme 1.4: Lack of knowledge

One participant had this to say:

‘My own view is that teenagers lack knowledge about use of contraceptives for example use of condom during intercourse and use of injectable or oral contraceptives. Other teenagers especially those who engage themselves for the first time don’t know the results of unprotected sex.’ (Participant 9)

Through probing, she continued to say:

‘Information regarding contraception is provided by the clinic staff only if you go there for family planning. In the rural villages no one comes to educate us on family planning.’ (Participant 10)

One of the major findings of this study was lack of information and education to high school learners regarding contraceptives to prevent unplanned pregnancies. There is need for collaborative efforts by healthcare providers from the clinics, school health nurses and private organisations to enhance health education talks including contraceptive use.

To prevent unplanned pregnancy, both boys and girls should have a good knowledge of conception and contraception, thorough discussion and commitment to assume shared responsibility of birth control, as well as constituent use of effective contraceptive methods. It implies a lack of knowledge and education regarding family planning use for teens at high schools and in the communities. Factors that result in an unplanned pregnancy leading to TOP may include no contraceptive use, contraceptive used wrongly, for example, forgetting to take oral contraceptive pill or wearing condom incorrectly, and contraceptive failure, for example, using contraceptive products beyond their expiry date.^20,21^

### Theme 2: Teenager’s fears regarding pregnancy

#### Sub-theme 2.1: Stigma, fear of parents and friends

One participant’s views regarding stigma were put forth as follows:

‘I opted for TOP because I was scared that my friends and teachers will laugh at me and might lose friendship. I also did it because I feared that my parents might chase me away from home and pressure from parents not being ready to be grandmother/father, and because parents do not like the person responsible for pregnancy.’ (Participant 12)

The study revealed fears of the stigma associated with pregnancy by high school teens. These teens are aware that falling pregnant as a teen at high school was not proper; hence, they chose TOP as a solution. Participants also feared their parents would be angry with them should they fall pregnant while at school as they were not yet ready to be grandparents. A gap was identified in this study to educate learners at high school regarding teenage pregnancy and contraceptives.

One of the main reasons teenage girls choose abortion is because they fear the stigma of being a pregnant teenager. While getting pregnant in high school is obviously not ideal and there are many hardships that come with the choice, the commendable thing to do is definitely to take off the problem by visiting a TOP clinic. Teens, in general, are more inclined to worry about what their parents, families and friends would say and think when they find out they are pregnant; therefore, they are scared that they would disappoint them and be in trouble.^5^ A social stigma was attached to pregnant, unmarried teens, and this increased the likelihood that unmarried teens opt for TOP. Recognition of this stigma usually led to fear, shame and embarrassment as well as decline in initial communication with family members. Fear of reaction of parents and friends increased secrecy.^21,22^

#### Sub-theme 2.2: Rape and incest

Some participants reported rape and incest as reflected by a participant who said:

‘I did it because I was raped by my uncle and my parents advised me to terminate pregnancy because they felt that the baby will be a curse to the family and can also be disabled.’ (Participant 15)

Another participant also said:

‘I decided to choose TOP because my sister’s husband was responsible for my pregnancy and I felt that there will be a conflict between me and my sister and other family members.’ (Participant 18)

The study findings reveal that some participants felt that falling pregnant as a teen because of rape and incest would bring curse to the family and even cause family disruptions. This implies that teenagers are aware and fear the consequences of rape and incest within families at their age. However, these teens would rather opt for TOP instead of giving birth to a disabled child in the family as a result of rape and incest. The findings of this study also revealed lack of education and knowledge regarding pregnancy and TOP at high schools in the rural communities.

Literature indicated that violent acts of rape and incest cause women to become pregnant on a daily basis. Not many people would argue that this causes psychologically harm to the victim. All over the world, including rural communities, children and teenagers are victims of rape and incest. Women are often in denial, too afraid to speak up or unaware they are pregnant. They may not know that emergency contraceptive is available to reduce incidents of becoming pregnant.^16^ Most teens are forced by their families to terminate the unborn babies associated with having to bring up a child born from incest or rape. In other incidences, babies are thrown away at birth because their parents do not allow them to raise the babies for fear that they might become a curse.^23^

#### Sub-theme 2.3: Fear of giving birth

A participant shared her views as follows:

‘My friend told me that giving birth is so painful in such a way that if I fall pregnant I might consider terminating the pregnancy.’ (Participant 21)

Poor communication regarding pregnancy, birth and TOP were found to be problematic among teens at the high schools. As a result, teenagers frighten each other regarding issues of childbirth. They instilled fear and anxiety associated with childbirth and the trauma associated with it. TOP was significantly found to be a more likely solution for high school teens than giving birth to a live baby. They opted for risky behaviours that threatened their quality of life. This implied that effective and extensive health education should be enforced among high school teens to enhance understanding around the issues of reproductive health. Hence, good communication, listening and counselling skills would be required to assist these rural high school teens to prevent unwanted pregnancies while still studying.

Fear of childbirth has become something of a modern day epidemic among teenagers. More than ever, teenagers are frightened about birth and what might possibly go wrong. Most teenagers feel a little anxious about giving birth, especially for the first time, but for some, childbirth can be intensely threatening and traumatic. Rather than looking forward to the arrival of their baby, they have a morbid fear of pregnancy and the birth process. Labour wards can feel like ‘torture chambers’ and they feel that their only option was to have TOP.^24,25^ Tokophobia can be caused by different factors including a fear of pain caused by past experience of a difficult childbirth, depression and even sexual trauma. Teens whose phobias occur after a traumatic delivery are likely to have experienced severe pain or tearing during birth or witness their baby in serious distress. As a result, when they become pregnant again, they cannot face the prospect of giving birth and they want to terminate.^26,27^

## Conclusion

The study results show that pregnant teenagers face various challenges that drive them to opt for TOP regardless of its consequences. TOP was viewed by teenagers as a means of safely getting rid of unwanted pregnancies as majority feared stigma from other peers and parents, lacked stable relationships, lacked knowledge on teen pregnancy and also feared giving birth at their age because they want to further their education.

### Recommendations

There are many factors that lead teenagers to opt for TOP; therefore, the following strategies to prevent TOP were suggested: discourage teenagers from engaging in sexual intercourse at an early age to prevent unwanted pregnancies that lead to TOP; providing health education regarding the use of different contraceptives; a lifestyle that leads teenagers to engage in sexual intercourse is discouraged (drugs and alcohol abuse) because it can lead to unwanted pregnancy and teenagers may opt for TOP; addressing the consequences and complications regarding TOP and conducting awareness campaign about TOP (at schools, clinic and community).
